# Serous Tubal Intraepithelial Carcinoma (STIC): A Review of the Literature on the Incidence at the Time of Prophylactic Surgery

**DOI:** 10.3390/diagnostics14222577

**Published:** 2024-11-16

**Authors:** Daniela Luvero, Roberto Angioli, Erika Notaro, Francesco Plotti, Corrado Terranova, Anna Maria Angioli, Asia Festa, Andi Stermasi, Serena Manco, Miriana Diserio, Roberto Montera

**Affiliations:** 1 Department of Gynecology, Fondazione Policlinico Universitario Campus Bio Medico, Via Alvaro del Portillo 200, 00128 Roma, Italy; 2 Research Unit of Gynecology, Department of Medicine and Surgery, Università Campus Bio Medico, Via Alvaro del Portillo 21, 00128 Roma, Italy

**Keywords:** STIC, prophylactic surgery, ovarian cancer, HGSC, BRCA mutations

## Abstract

Background: Serous tubal intraepithelial carcinoma (STIC) is an early-stage cancerous lesion found in the fallopian tubes, often at the fimbrial end. It is strongly associated with high-grade serous carcinoma (HGSC), a highly aggressive type of ovarian cancer. STIC is considered a precursor to many HGSC cases, originating in the fallopian tubes. Its development is frequently linked to mutations in the TP53 gene, leading to the formation of a p53 signature, an early abnormality that may progress to HGSC. This signature is more common in BRCA mutation carriers, explaining the higher incidence of STIC in this group. The aim of this review is to evaluate the literature on the incidence of serous tubal intraepithelial carcinoma in patients (both BRCA-positive and BRCA-negative) undergoing preventive salpingo-oophorectomy, analysing the available data and identifying associations between specific characteristics and the onset of STIC. Methods: A comprehensive review of the literature from 2016 to 2023 was conducted using PubMed, focusing on studies analysing the incidence of STIC in BRCA-positive patients undergoing preventive salpingo-oophorectomy. Data on patient characteristics, interventions, outcomes, and incidence of STIC were extracted and analysed. Results: Nine international studies were included in the review, reporting varying incidences of STIC among patients undergoing salpingo-oophorectomy. The overall incidence of STIC in all the women included in the studies was 7.31%, while that in the BRCA-mutated women was approximately 6.08%. Notably, the presence of the TP53 signature was significantly associated with the occurrence of STIC. Conclusions: The etiopathogenesis of STIC involves complex interactions between genetic, environmental, and molecular factors. Further research is needed to fully understand its mechanisms and identify additional risk factors beyond BRCA mutations. Establishing a national database of STIC cases could facilitate future research and improve patient outcomes.

## 1. Introduction

Serous tubal intraepithelial carcinoma (STIC) is a type of pre-invasive or early-stage cancerous lesion found in the epithelial cells lining the fallopian tubes, more specifically at the fimbrial end. STIC is often associated with high-grade serous carcinoma (HGSC), an aggressive type of ovarian cancer (OC), which is the primary reason for mortality attributed to gynaecologic malignancies, resulting in approximately 12,810 deaths annually in the United States [1]. Most cases—over 70%—of HGSC are diagnosed at FIGO stage III or IV due to vague symptoms in the early stages of the disease and the absence of reliable screening methods. Despite advancements in treatment, the mortality rate remains high, with a five-year overall survival for advanced-stage disease ranging from just 20–40% [2].

It is believed that many cases of high-grade serous ovarian cancer may originate in the fallopian tubes, with STIC representing the precursor lesion [3,4,5,6]; the average duration of STIC progression to HGSC is 6.5 years [7].

To support the hypothesis that the fallopian tubes are the primary site of origin, rather than being secondarily involved, and that STIC formation is an early step in cancer development, telomere length was examined in different studies. The analysis showed telomere shortening in STIC compared to the surrounding normal tubal epithelium. Telomere shortening is a well-known genetic feature of cancer. Notably, the telomeres in STIC patients were even shorter than those in patients suffering from the associated HGSC, reinforcing the idea that STIC formation is an early event in the carcinogenesis process [8,9].

Further evidence supporting the idea that HGSC originates from STIC comes from mouse model studies, which demonstrated that removing the fallopian tubes, but not the ovaries, effectively prevents the formation of HGSC [10,11,12].

Nowadays, the prevailing hypothesis concerning the initiation of STIC suggests that mutations in the TP53 gene within the fallopian tube’s epithelium trigger the formation of the so-called “p53 signature”. It is proposed that the junction between the fallopian tube epithelium and ovarian mesothelium could be a potential site for cancer development, like other junctional areas such as the uterine, cervical, gastroesophageal, or anorectal regions. The fimbrial end’s exposure to locally elevated inflammatory cytokines during ovulation may initiate the formation of precursor lesions, leading to a malignant transformation. This process likely involves the selection of p53 mutations in epithelial cells, which are then clonally expanded [13,14,15]. The exact process by which fallopian tube cells implant into the ovary is still unclear. However, it is believed that the rupture of the dominant follicle during ovulation may expose the ovarian stroma to the fimbrial epithelium. This exposure could allow precursor lesions to attach to the ovarian surface or penetrate the ovarian stroma [16,17,18,19,20]. The p53 signature is identified by abnormal p53 staining in at least 12 consecutive, normal-appearing tubal epithelial cells. It is the early lesion leading to STIC, characterised by enlarged nuclei, increased atypia, loss of polarity, abnormal p53 expression, and higher proliferation, which can progress to high-grade serous ovarian cancer [21]. It represents the earliest identified DNA sequence alteration in STIC and is nearly ubiquitous in invasive HGSC, affecting the ovaries and peritoneum [22]. The distal fallopian tube epithelia of BRCA mutation carriers show several transcriptional differences compared to the tubal epithelia of unaffected women: the presence of the p53 signature is higher in these women, explaining the frequent finding of STIC in their cases [23]. BRCA mutations increase a woman’s risk of developing ovarian cancer, in fact around 17–20% of OC is due to faulty BRCA genes.

According to the literature, the incidence of STIC in BRCA-positive patients is heterogeneous and is reported to be around 7.31% [7,21,24,25,26,27,28,29,30]. While current evidence suggests that STIC could act as a precursor for ovarian carcinoma in high-risk women with inherited BRCA1 or BRCA2 mutations, the clinical significance of STIC is not as well-defined in women from the general population, where 85–90% of all HGSC originate [31]. Many studies from the past suggested that STIC is not only linked to BRCA mutations, in fact, Kindelberger et al. characterised a cohort of women with ovarian carcinoma, revealing that almost none of them carried BRCA germline mutations. In 35–50% of these cases, the simultaneous detection of STIC occurred alongside pelvic serous carcinoma [32].

STIC development is linked to BRCA1 and BRCA2 mutations. The American College of Obstetricians and Gynecologists (ACOG) and the Society of Gynecologic Oncology (SGO) recommend that women with these mutations consider bilateral salpingo-oophorectomy (BSO) after childbearing to reduce their ovarian cancer risk [33]. The benefits of prophylactic BSO for women at high genetic risk are well-established, reducing ovarian cancer risk by over 80% in BRCA mutation carriers, as shown in studies by Powell and Kwon, and a meta-analysis of ten studies [34,35,36]. Additionally, a recent study involving approximately 5800 mutation carriers corroborates this finding, showing a similar decline in ovarian cancer risk and, notably, a 77% decrease in overall mortality by age 70 [37]. The remaining risk for ovarian cancer is attributed to undetected cancers at the time of surgery or the later emergence of primary peritoneal cancer. Furthermore, BSO has been linked to a lowered risk of breast cancer, particularly in pre-menopausal women with BRCA2 mutations [38,39]. It is recommended that prophylactic BSO be conducted between the ages of 35 and 40, and after completing childbearing, for all women with Hereditary Breast and/or Ovarian Cancer Syndrome.

However, adherence to this suggestion has been somewhat limited, primarily due to apprehensions regarding the early onset of menopause [40]. In fact, undergoing oophorectomy in pre-menopausal women elevates the risk of cardiovascular morbidity, osteoporosis, and symptoms associated with endocrine changes, such as hot flashes, as well as challenges with sexual function [41]. Salpingectomy appears safer and less harmful than salpingo-oophorectomy [42,43,44]. Removing just the fallopian tubes during pelvic or abdominal surgery for a non-cancerous condition (known as opportunistic salpingectomy) seems to be a promising strategy for the primary prevention of HGSC but is now recommended for women that have no BRCA mutations and have completed their families [45]. Significant efforts have been made to develop surveillance methods, particularly for patients at a high risk for ovarian cancer, which is often undetected until the advanced stage with poor outcomes. The early detection of lesions like STIC is crucial for managing ovarian and fallopian tube cancers. Given the link between BRCA1/BRCA2 mutations and STIC, several studies have examined STIC incidence in fallopian tubes removed during preventive salpingo-oophorectomy in these women. This review includes recent studies on the STIC incidence in BRCA-mutated patients undergoing BSO, analyses shared patient characteristics to highlight molecular aspects, and identifies potential STIC and ovarian cancer risk factors.

## 2. Materials and Methods

This review was initiated by conducting comprehensive literature searches on PubMed, encompassing papers published from January 2016 up to December 2023. The search terms employed were carefully selected, including specific phrases such as “serous tubal intraepithelial carcinoma”, “STIC”, “BRCA1”, “BRCA2”, and “prophylactic salpingo-oophorectomy”.

Papers were included based on the following criteria:

1. Published between January 2016 and December 2023.
2. Focused on STIC, BRCA1/BRCA2 mutations, and prophylactic salpingo-oophorectomy.
3. Included data on patient characteristics (possible genetic mutations, age, history of breast cancer).
4. Provided detailed information on interventions such as screening, risk-reducing salpingo-oophorectomy, or bilateral salpingectomy with delayed oophorectomy.

Papers were excluded based on the following:

1. Published prior to January 2016.
2. Did not specifically address STIC, BRCA1/BRCA2 mutations or prophylactic surgeries.
3. Lacked patient characteristics, interventions, or outcome data relevant to the scope of this review.
4. Articles, case reports, or non-original research were not peer reviewed.

In total, we found 17 articles, selected nine articles following the inclusion criteria, and excluded eight articles. 

Once identified, relevant data from these papers were systematically extracted and organised based on key factors such as patient characteristics, details of interventions, and crucial outcomes. Special attention was given to gathering information on the presence of BRCA1 or BRCA2 mutations, the manifestation of the TP53 signature, and various aspects of patient management, such as screening, risk-reducing salpingo-oophorectomy, or bilateral salpingectomy with delayed oophorectomy. Additional details sought included the average age at surgery (if applicable), history of breast cancer, and use of tamoxifen.

The final step involved a comprehensive analysis, including the quantification of cases of STIC, ovarian carcinoma, or tubal carcinoma across all papers incorporated in the review.

## 3. Results

According to the inclusion criteria, we included nine international studies sourced from 2016 to 2023, specifically: one from 2016 (Zakhour et al.), two from 2018 (Nebgen et al. and Minig et al.), one from 2019 (Blok et al.), one from 2020 (Rudaitis et al.), one from 2021 (Boerner et al.), two from 2022 (Sina et al. and Loizzi et al.), and one from 2023 (Byun et al.). See Table 1 for details of the studies [7,21,24,25,26,27,28,29,30].

In 2016, Zakhour et al.’s study involved 257 women undergoing risk-reducing salpingo-oophorectomy. Among these women, 148 had a mutation in the BRCA1 gene, 98 had a mutation in the BRCA2 gene, six had unspecified BRCA mutations, and five had mutations in both BRCA1 and BRCA2. Occult carcinoma, meaning cancer that wasn’t clinically detectable before surgery, was found in 14 patients. This included nine cases of STIC with an incidence rate of 3.5%, three cases of tubal cancer, one case of ovarian cancer, and one case of endometrial cancer. Additionally, the study noted that 110 women had been previously diagnosed with breast cancer before undergoing surgery, with 74 of them using tamoxifen [30].

In the prospective, multicentre, non-randomised pilot study conducted by Nebgen et al., a total of 43 patients were recruited, of which 16 had BRCA1 mutations and 27 had BRCA2 mutations. Among these, 12 opted for surveillance, 12 opted for preventive salpingo-oophorectomy, and 19 opted for bilateral salpingectomy, deferring oophorectomy until a later time. Only one case of STIC was found in this study (incidence of 2.3%) [24].

In Minig et al.’s retrospective multicentric observational study, 359 BRCA pathogenic mutation carriers who had undergone a risk-reducing salpingo-oophorectomy were evaluated. STIC was diagnosed in three patients, with an incidence of 0.8%. Also, one case of ovarian cancer was found [26].

Blok et al.’s study involved 527 patients who underwent risk-reducing surgery to remove their fallopian tubes and ovaries. Among these patients, 358 had a BRCA1 mutation, 167 had a BRCA2 mutation, and two had mutations in both BRCA1 and BRCA2. Additionally, 202 patients had previously been diagnosed with breast cancer, with 141 being carriers of the BRCA1 mutation and 61 carrying the BRCA2 mutation. Four cases showed the presence of STIC, with an incidence of 0.8%. Also, seven cases of tubal carcinoma were reported [28].

Rudaitis et al. conducted a study involving 564 women who tested positive for new germline BRCA1/2 mutations. This cohort included healthy carriers with a family history of ovarian or breast cancer (178 individuals), those previously diagnosed with breast cancer (260 individuals), and ovarian cancer patients (126 individuals). As part of their management for reducing ovarian cancer risk, 71 eligible women underwent laparoscopic prophylactic BSO. Among them, seven were found to have STIC, with an incidence rate of 9.5%. Four new cases of ovarian cancer were also identified among these women [29].

The analysis conducted by Boerner et al. aimed to assess the clinical relevance and genomic correlations of concurrent STIC with high-grade serous ovarian cancer in a cohort of 306 women undergoing primary debulking surgery. Among these cases, STIC was identified in 87 instances, representing an incidence of 28%. Additionally, a significant finding was the presence of the TP53 signature in the fallopian tube epithelium of 144 women (it’s noteworthy that targeted DNA sequencing was conducted on samples from 147 patients out of 306) [25].

A total of 194 women with a known germline pathogenic mutation of BRCA1 or BRCA2 genes undergoing risk-reducing salpingo-oophorectomy were considered in Sina et al.’s analysis. A total of 102 women had mutations of BRCA1 and 92 had mutations of BRCA2. A total of 15 cases of STIC were found among them (incidence of 7.73%). From this same analysis, other interesting data emerged: out of the 194 cases, 122 women had a history of previous breast cancer, and 45 had undergone tamoxifen therapy. In addition to the 15 cases of STIC, five cases of ovarian carcinoma were identified. The presence of a TP53 signature was detected within the fallopian tube epithelium in 36 cases [21].

Risk-reducing surgery was performed in 100 patients carrying BRCA mutations (in particular, 59 had BRCA1 mutations and 41 had BRCA2 mutations) in Loizzi at al.’s prospective multicentric study. Among them, 58 patients had previous history of breast cancer. STIC was found in two cases (incidence of 2%) and ovarian cancer in one case [27].

According to Byun et al.’s analysis conducted on 76 women with high-grade serous ovarian cancer undergoing BSO, 11 cases of STIC were reported, with a total incidence of STIC of 14.47%. Out of all the cases, 17 had unknown BRCA mutation status, leaving 59 cases analysed for STIC based on BRCA mutation status. BRCA mutations were detected in 16.9% of patients diagnosed with high-grade serous ovarian cancer. Among the 10 cases with BRCA mutations, STIC was present in three cases (30% incidence), while among the 10 cases without BRCA mutations, STIC was observed in seven cases (14.3% incidence) [7].

The total number of women included in the studies we considered is 2503, while the number who underwent salpingo-oophorectomy is 1902. The total number of STIC cases reported in this last group is 139. As a result, the total incidence of serous tubal intraepithelial carcinoma in women undergoing salpingo-oophorectomy in the most recent literature is approximately 7.31% [7,21,24,25,26,27,28,29,30].

We used data on STIC cases among women with BRCA1 and BRCA2 mutations across multiple studies. Zakhour et al. identified 148 BRCA1 cases, 98 BRCA2 cases, five BRCA1/2 cases, six unspecified BRCA cases and nine STIC cases; Negben et al. reported 16 BRCA1 cases, 27 BRCA2 cases, and one STIC case; Minig et al. observed 359 unspecified BRCA cases and three STIC cases; Blok et al. found 358 BRCA1 cases, 167 BRCA2 cases, and four STIC cases; Rudaitis et al. noted 564 unspecified BRCA cases and seven STIC cases; Sina et al. recorded 102 BRCA1 cases, 92 BRCA2 cases, and 15 STIC cases; Loizzi et al. documented 59 BRCA1 cases, 41 BRCA2 cases, and two STIC cases; Byun et al. included 10 unspecified BRCA cases and three STIC cases. Summing up the numbers, we found a total of 683 BRCA1 cases, 425 BRCA2 cases, and 1046 BRCA1/2 or unspecified BRCA cases across all studies. This adds up to 2154 cases of BRCA-mutated women. Among them, 131 STIC cases were reported, resulting in an overall incidence of 6.08% among all BRCA-mutated women studied [7,21,24,25,26,27,28,29,30].

In the context of this comprehensive review, it is noteworthy that two of the nine studies scrutinised the presence of the TP53 signature within the tubal epithelium of patients undergoing salpingo-oophorectomy; in the analysis by Sina et al., among 194 patients subjected to surgery, 36 exhibited the TP53 signature, representing an incidence of 18.56%. Of these cases, 15 manifested the presence of STIC, resulting in an incidence of 41.67% for STIC among patients with the TP53 signature [21]; in Boerner et al.’s investigation, only 147 out of 306 enrolled patients were investigated for the presence of the TP53 signature, and indeed a notable 98% (144 patients) tested positive for the genomic imprint. STIC was identified in 87 of these patients, yielding an incidence of 60.42% for STIC among those presenting the TP53 signature [25].

In five out of the nine studies, additional information was examined, including prior breast cancer history and potential tamoxifen usage. In the study conducted by Sina et al., among the 194 enrolled patients, 122 had a history of breast cancer, with 45 of them having undergone prior tamoxifen therapy [21]. Among the 257 patients studied by Zakhour et al., 110 had a history of breast cancer, with 74 of them having undergone previous tamoxifen therapy [30]. Loizzi et al.’s analysis revealed that 58 out of 100 patients had a history of breast cancer [27]. In the study by Blok et al., 202 out of the 527 enrolled patients had a history of breast cancer [28]. According to Rudaitis et al., among 564 patients, 260 had a history of breast cancer [29]. Data regarding any instances of patients undergoing tamoxifen therapy are not available for the latter three cases.

## 4. Discussion

Individuals harbouring BRCA mutations face notably elevated risks of breast and ovarian cancers. Furthermore, as noted in our review, those with BRCA mutations also confront heightened susceptibility to pre-tumoural lesions such as STIC. However, advancements in proactive interventions such as chemoprevention, prophylactic surgeries, and targeted therapies have demonstrated outcomes comparable to non-carriers, underscoring the significance of early identification [46]. The timely recognition of potential mutation carriers enables the implementation of comprehensive measures encompassing genetic counselling, testing, screening, and prevention strategies. This approach not only optimises patient care but also empowers individuals with informed choices, ultimately enhancing cancer management and prognosis.

It is crucial to understand the pathogenetic mechanisms underlying the mutations in the BRCA1 and BRCA2 genes and how these are qualitatively and quantitatively linked to the risk of developing pre-tumour lesions like STIC and gynaecological cancers. Having a BRCA1 mutation significantly elevates a woman’s chances of experiencing breast cancer throughout her lifetime, with a risk as high as 85%, and increases the likelihood of ovarian cancer to approximately 40–50%. Conversely, for those with a BRCA2 mutation, the risk of breast cancer ranges from 40–45%, while the risk of ovarian cancer is between 15–30% [47]. BRCA1 and BRCA2 genes are typically classified among DNA repair genes, playing a regulatory role in the cell cycle by encoding proteins involved in responding to DNA damage, and hence, acting as tumour suppressor genes. These genes are also implicated in synthesising multiprotein complexes that regulate DNA synthesis transcriptionally and in recognising and correcting double-stranded breaks in certain types of DNA damage. Mutations in these DNA repair genes lead to functional deficiencies, impairing DNA repair and causing irregularities in DNA synthesis, primarily manifesting as point mutations or deletion/insertion mutations. Because of these mutations, p53-dependent DNA breakdown is triggered, potentially resulting in cell cycle arrest and apoptosis [48]. This leads to the p53 signature, which, as stated before, is the preliminary lesion leading to STIC. More specifically, compared to those without mutations, the cells lining the fallopian tubes of women, particularly those with BRCA1 mutations, show higher levels of certain proteins, like NAMPT, which helps with cell stress, and C/EBP-δ, which is involved in inflammation, DNA repair, and the development of tumours. Also, markers of DNA damage and repair, like P53 and γ-H2A, are more active in the fallopian tube and ovarian surface cells of BRCA1/2 mutation carriers than in those without mutations. When women ovulate, their fallopian tube ends are exposed to substances that can damage DNA, and this process seems to affect women with BRCA mutations more [23]. 

Approximately 314,000 new cases of ovarian cancer occurred in 2020 [49]. Despite its increasing incidence, ovarian cancer remains a rare condition. STIC, which represents the precursor to ovarian cancer, is even rarer: it is noteworthy that the incidence of STIC in BRCA-mutated women undergoing preventive salpingo-oophorectomy ranges from 0.6 to 7%, according to an exhaustive search of the literature [50], and is 7.31% according to our review, which gathers the most recent data. 

Based on the importance of the molecular aspects, the patients’ genetic profile, and family history, the management of ovarian cancer should involve a dedicated multidisciplinary team, including gynaecologic oncologists, gynaecologic surgeons, radiation oncologists, radiologists, geneticists, psychologists, and pathologists. It would be desirable to always have a specialised pathology team dedicated to ovarian cancer research, capable of promptly identifying precancerous lesions such as STIC, thus providing precise histological diagnoses. In this multidisciplinary team, radiologists play a critical role: using imaging modalities such as ultrasound, CT, and MRI, radiologists assess the tumour size, location, and its metastasis, while differentiating malignant from benign masses, thereby helping to avoid unnecessary surgery and tailoring treatment options. Imaging also facilitates the evaluation of non-surgical treatments, such as chemotherapy, targeted therapy, and hormone therapy by tracking tumour response and progression. Additionally, radiologists may perform image-guided biopsies, enabling minimally invasive sampling from otherwise difficult-to-access tumours, reducing patient discomfort and procedural risks. 

Pathologists are another essential figure in staging ovarian cancer and guiding treatment through molecular testing, identifying mutations that influence therapy, such as BRCA with PARP inhibitor treatment. By tracking molecular markers, they support personalised treatment and monitor disease progression, enabling adjustments to therapy as needed. Pathological analysis is vital from diagnosis through treatment, ensuring accurate staging and management. 

Cancer diagnosis, especially ovarian cancer with its challenging prognosis, can be overwhelming. Psychologists provide vital support, helping patients to manage stress, understand the genetic risks, and cope with genetic information, which is not necessarily predictive of mortality but may lead to proactive health measures. Through counselling, they also address psychological barriers like depression or treatment fatigue, aiding patients in adhering to treatment and follow-up care.

From the results of our study, it is evident that there is a close correlation between breast cancer history and the development of STIC. This is not surprising, as breast cancer and ovarian cancer are known to be part of the Hereditary Breast and Ovarian Cancer Syndrome (HBOC), which affects BRCA-mutated individuals. What emerges from two studies (Sina et al., Zakhour et al.) is that there is a subset of patients with a history of breast cancer who have developed STIC and who, previously, underwent tamoxifen therapy [21,30]. This information might suggest a correlation between tamoxifen use and the onset of STIC, but the limited amount of information regarding this link does not provide additional insights. It is likely that the development of STIC is simply correlated with HBOC, but it would be interesting to pose questions to lie the groundwork for future studies that could also investigate seemingly secondary factors as potential contributors to the development of STIC and, consequently, ovarian cancer.

The strength of our review is attributable to the fact that it examines the most recent literature concerning the incidence of STIC in prophylactic salpingo-oophorectomies in BRCA mutation carriers. Also, the sources utilised are reliable and up-to-date, and the data presented have been carefully analysed. However, the weaknesses of this review are primarily based on the rarity of the condition and the limited number of studies specifically focused on our research. Furthermore, the heterogeneity of factors considered in each study may pose a limitation in the analysis of the results.

## 5. Conclusions

The etiopathogenesis of STIC is still a topic under study requiring further understanding. There are several theories attempting to explain its development, including those related to specific genetic mutations (such as those in the BRCA1 and 2 genes, which are associated with an increased risk of developing STIC) and those concerning the involvement of the microenvironment of the fallopian tubes, which, when subjected to the action of hormones and/or chronic inflammation, may play a significant role in the process leading to STIC. In summary, the etiopathogenesis of STIC is complex and involves a combination of genetic, environmental, and molecular factors. Further research is needed to fully understand the mechanisms that lead to its formation and progression. 

Understanding the etiopathogenesis of STIC is important because it could potentially lead to the prevention of ovarian cancer cases that originate from it, which could be detected early through more in-depth analyses of the various steps involved in neoplasia development.

To lie the groundwork for future research on STIC, it would first be necessary to have a larger case series to analyse, perhaps with a greater number of multicentric studies focussed not only on identifying the incidence of STIC but also on identifying risk factors and molecular characteristics in addition to BRCA mutations, which may contribute to predisposing the development of this precancerous lesion; a desirable outcome would be the identification of molecular biomarkers (urine, blood, or others) that mark the risk of developing ovarian cancer without the analysis of pathological tissue. 

Another useful intervention that could be implemented to thoroughly study STIC would be to create a national and shared database of STIC cases to be able, in the future, to track patients who had this lesion before developing ovarian cancer to better understand the clinical advancement of the disease.

## Figures and Tables

**Table 1:** Studies included.

Author	Journal	Number of Patients	BRCA1	BRCA2	BRCA1 and 2 + Not Specified	TP53+	Average Age at Surgery	Surveillance	Salpingo-oophorectomy	Bilateral Salpingectomy with Delayed Oophorectomy	Previous Breast Cancer	Previous Tamoxifen Use	STIC (New Diagnosis)	Ovarian Carcinoma (New Diagnosis)	Tubal Carcinoma (New Diagnosis)	% (Incidence of STIC)
Nebgen [24]	Gynecologic oncology	43	16	27				12	12	19			1	0	0	2.3% in BRCA+
Sina [21]	International journal of gynecological oncology	271 (**194** + 77)	102	92		**36** + 12	50		194		122	45	15 + 0	5		7.73% in BRCA+, 0% in non-mutated
Byun [7]	Taiwanese Journal of Obstetrics & Gynecology	76			10		56		76				11 (3 in BRCA+, 7 in non mutated)	76		30% in BRCA+, 14.3% in non-mutated
Boerner [25]	Gynecol Oncol.	306				144 (of 147 out of 306)			306				87			28%
Minig [26]	Federation of Spanish Oncology Societies and of the National Cancer Institute of Mexico	359			359				359				3	1		0.8% in BRCA+
Loizzi [27]	Acta Biomedica: Atenei Parmensis	100	59	41	100		51		100		58		2	1		2% in BRCA+
Blok [28]	Gynecologic oncology	527	358	167	2				527		202		4		7	0.8% in BRCA+
Rudaitis [29]	European journal of obstetrics, gynecology, and reproductive biology	564			564		46.5		71		260		7	4		9.85% in BRCA+
Zakhour [30]	Gynecologic oncology	257	148	98	5 + 6				257		110	74	9	1	3	3.5% in BRCA+

## Data Availability

The original contributions presented in this study are included in this article. Further inquiries can be directed to the corresponding author.
